# First molecular characterization of capsule expression and antibiotic susceptibility profile of *Staphylococcus aureus* isolates from bovine mastitis in Jordan

**DOI:** 10.14202/vetworld.2022.2269-2274

**Published:** 2022-09-23

**Authors:** Mohammad Hamdi Gharaibeh, Luay F. Abu-Qatouseh

**Affiliations:** 1 Department of Basic Veterinary Medical Science, Faculty of Veterinary Medicine, Jordan University of Science and Technology, P.O. Box 3030 Irbid, 22110 Jordan; 2 Department of Pharmacology and Biomedical Sciences, Faculty of Pharmacy, University of Petra, Amman, Jordan

**Keywords:** antibiotic susceptibility, bovine mastitis, capsule genotypes, polymerase chain reaction, *Staphylococcus aureus*

## Abstract

**Background and Aim:**

Bovine mastitis has long been considered the most important cause of economic losses in the dairy industry. *Staphylococcus aureus* is the most frequently isolated pathogen from bovine mastitis cases worldwide. Capsular polysaccharides (CPs) of serotype 5 (CP5) or serotype 8 (CP8) are the most prevalent capsule genotypes related to infections associated with *S. aureus* in humans. However, a variety of CPs has been reported in ruminants and other hosts. Information regarding the relationship between genotypic and phenotypic capsule variation and bovine mastitis in Jordan is scarce. Thus, we aimed to determine the prevalence of *S. aureus* capsule genotypes CP5 and CP8 in milk from bovine mastitis cases and the antimicrobial susceptibility profile of the recovered isolates in 27 dairy farms in Jordan.

**Materials and Methods:**

*Staphylococcus aureus* strains were isolated from bovine mastitis cases in two districts of Jordan. All *S. aureus* isolates were initially identified using conventional biochemical and microbiological methods. Subsequently, confirmation of the identity of *S. aureus* was performed by polymerase chain reaction (PCR) targeting *nuc* gene. Capsule polysaccharide typing was performed by PCR specific for CP5 and CP8. In addition, we assessed the antibiotic susceptibility profile of *S. aureus* isolates against commonly used antimicrobials by the disk diffusion method according to Clinical and Laboratory Standards Institute guidelines.

**Results:**

We collected 148 clinical isolates of *S. aureus* from bovine mastitis cases in the Zarqa (67.6%, n = 100) and Irbid (32.4%, n = 48) districts. Most isolates possessed capsule genotypes (91.3%), predominantly CP8 (88.6%). Only 8.7% of the isolates were nontypeable by PCR. In addition, we found statistically significant differences between the geographical region and the status of methicillin-resistant capsule genotypes (p < 0.05). The rates of resistance to β-lactam, macrolide, and fluoroquinolone antibiotics were very low, but resistance to tetracyclines was considerably high (22.3%). Significantly, mastitis isolates from Irbid showed a higher rate of resistance to ciprofloxacin (8.3% vs. 0%), while isolates from Zarqa showed a significantly higher rate of resistance to gentamicin (12.0% vs. 6.2%).

**Conclusion:**

We established associations between capsule genotypes and antimicrobial resistance and the pathogenic behavior of *S. aureus* isolated from bovine mastitis cases. Further studies are necessary to fully elucidate the role and mechanisms of capsular expression in the epidemiological and molecular variability of *S. aureus* in bovine mastitis.

## Introduction

Bovine mastitis (inflammation of the mammary gland) is a serious issue that causes significant economic losses to the dairy industry [1], as well as losses in the bovine, caprine, and swine industries [2]. There are 137 microorganisms that can cause bovine mastitis [3], of which *Staphylococcus aureus* is one of the most common etiological agents associated with both clinical and subclinical mastitis worldwide and in Jordan [3, 4]. *Staphylococcus aureus* bovine mastitis can be controlled by milking-time hygiene, culling chronically infected animals, and antibiotic treatment [5]. However, the increased spread of antibiotic resistance [6] and the development of strains of multidrug-resistant *S. aureus* [7] have made infection control incredibly challenging. Due to these limitations, a possible approach to preventing and controlling bovine mastitis is the development of protective vaccines against this disease [8, 9].

The development of an effective vaccine requires a detailed description of the key virulence factors involved in the infection. *Staphylococcus aureus* possesses numerous virulence factors that affect the pathogenesis of bovine mastitis, such as adhesins, capsular polysaccharides (CPs), exoenzymes, and toxins [10]. In particular, the *S. aureus* capsule enhances microbial virulence by evading phagocytosis [11]. Of the 11 capsular serotypes (CPs) discovered based on polyclonal antisera, only four (CP1, CP2, CP5, and CP8) have been fully characterized. Among them, CP5 and CP8 are the most common serotypes in human and bovine infections. *Staphylococcus aureus* isolates that do not react with antibodies to CP1, CP2, CP5, and CP8 are defined as nontypeable (NT) [12]. CP5 and CP8 are key in vaccine research and development because antibodies against CPs opsonize *S. aureus*, enhancing killing by neutrophil phagocytosis [9–11, 13]. Information concerning the geographical distribution of capsular serotypes and genotypes is crucial for the rational design and use of vaccines against *S. aureus* mastitis in the future. However, data on CP5 and CP8 in bovine mastitis are scarce. Geographic variations in the rates of bovine mastitis have been observed worldwide, some of which are related to capsular genotypes. For example, 90% of 210 *S. aureus* bovine isolates from Japan were identified as CP5 or CP8 [14], while only 14% of isolates from Argentina were CP5 or CP8 [15]. Further evidence of differences in capsular genotypes was observed in bovine mastitis cases from Europe and the United States, where 42% and 87% of cases, respectively, were serotypeable isolates [16].

To the best of our knowledge, this is the first study to determine the prevalence of *S. aureus* CP5 and CP8 serotypes in milk from bovine mastitis cases in Jordan. We also investigated the susceptibility profiles of the isolates to commonly used antimicrobial agents.

## Materials and Methods

### Ethical approval

The study protocol was approved by the Jordan University of Science and Technology (JUST) Animal Care and Use Committee (ACUC, Project # 163/2015).

### Study period and location

**Figure-1:**
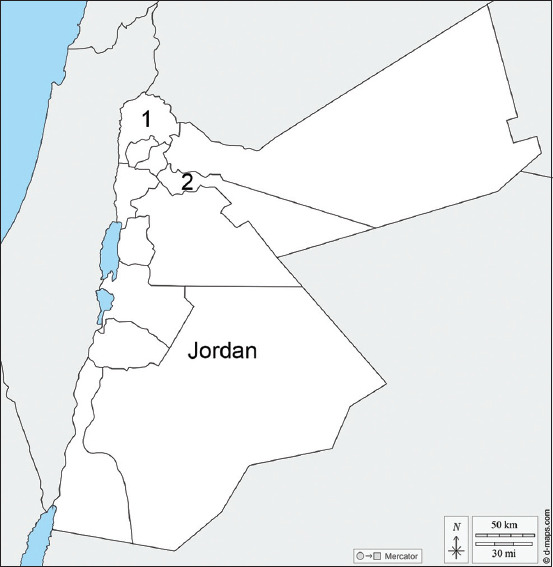
Geographic distribution of the bovine milk samples. A total of 148 subclinical mastitis milk samples were collected from 27 farms in Jordan; (1: Irbid, n = 48), and (2: Zarqa (Dhlail region, n = 100) [Source: https://d-maps.com/carte.php?num_car=5402&lang=en].

The study was conducted from April 2016 to November 2018. The milk samples were collected from 27 farms in two districts in Jordan: Irbid and Zarqa (Dhlail), which represented the North and Middle parts of Jordan, Figure-1. The bacteriological and molecular works were conducted in the Laboratory of Microbiology, Faculty of Veterinary Medicine, Jordan University of Science and Technology, and the University of Petra Pharmaceutical Center.

### Collection and identification of *S. aureus* mastitis isolates

We collected and tested milk for subclinical mastitis using the California Mastitis Test protocol according to National Mastitis Council [17]. Briefly, the teats from individual mammary glands were dipped into an iodine solution and dried with a towel impregnated with 70% alcohol. Then, a few strips of foremilk were discarded before collecting milk samples of approximately 10 mL in 15 mL sterile falcon tubes that were immediately placed in an icebox and sent to the laboratory that same day. We performed a primary culture of each milk sample (10 mL) on 5% sheep blood agar plates incubated aerobically at 37°C for 24 h. Presumptive *S. aureus* isolates were identified based on the following characteristics: Gram-positive cocci, blood agar hemolysis, and positive for catalase and coagulase. Only those milk samples that were positive for *S. aureus* were used in the next steps.

### DNA extraction

We extracted DNA from four to five colonies of fresh subcultured *S. aureus* isolates on nutrient agar plates using a QIAamp DNA Mini Kit (Qiagen, Hilden, Germany) in accordance with the manufacturer’s instructions except for some modifications of the preincubation step with lysozyme (10 mg/mL) and lysostaphin (10 μg/mL) for 45 min. The extracted DNA was stored at −20°C until further use.

### Molecular characterization of capsule genotypes

First, all isolates were confirmed as *S. aureus* by the presence of *nuc*, which encodes thermonuclease, and were tested for the presence of *mecA*, which encodes methicillin resistance, as previously described by Alekish *et al*. [18]. *Staphylococcus* *aureus* strain ATCC 29213 (American Type Culture Collection, Manassas, VA, USA) was used as the positive control. Then, the expression of CP5 or CP8 was determined by polymerase chain reaction (PCR) as described by Sordelli *et al*. [15]. Primer pairs targeting the CP5 gene sequence (Cap5 F: 5'–GAA AGT GAA CGA TTA GTA GAA–3' and Cap5 R: 5'–GTA CGA AGC GTT TTG ATA GTT–3') and the CP8 gene sequence (Cap8 F: 5'–GTG GGA TTT TTG TAG CTT TT–3' and Cap8 R: 5'–CGC CTC GCT ATA TGA ACT AT–3') were used. The polymerase chain reaction was performed on a MyCycler Thermal Cycler (BioRad, Hercules, CA, USA) under the following conditions: initial denaturation at 94°C; 30 cycles of 30 s of denaturation at 94°C, 30 s of annealing at 45°C, and 2 min of extension at 72°C; and final extension at 72°C for 5 min. The PCR products were analyzed following agarose gel electrophoresis and staining with ethidium bromide.

### Antimicrobial susceptibility tests using the standard disc diffusion method

We performed disk diffusion tests for *S. aureus* isolates according to the Clinical and Laboratory Standards Institute guidelines [19] with slight modifications as previously described by Gharaibeh *et al*. [20]. The isolates were tested for resistance to ten antimicrobial agents using antibiotic discs (Oxoid, England), including erythromycin (E, 15 μg), clindamycin (DA, 10 μg), cefoxitin (FOX, 30 μg), trimethoprim/sulfamethoxazole (SXT, 25 μg), doxycycline (DOX, 30 μg), tetracycline (TE, 30 μg), chloramphenicol (C, 30 μg), ciprofloxacin (CIP, 5 μg), ceftazidime (CZ, 30 μg), and gentamicin (CN, 10 μg).

### Statistical analysis

Capsule gene frequencies were calculated as categorical (binary) variables using Microsoft Excel. Chi-square test was used to compare the distribution of CP5, CP8, and NT strains among different groups of antimicrobial agents using the statistical package for the social sciences (SPSS) version 17.0 (SPSS Inc., Chicago, IL, USA).

## Results

### Capsular polysaccharide typing and prevalence among *S. aureus* isolates

We cultured 148 *S. aureus* isolates from milk samples collected from two distinct geographical areas in Jordan: Zarqa and Irbid (Figure-1). The distribution of capsule genotypes CP8 and CP5 was 131 (88.5%) and 4 (2.7%), respectively (Table-1). In addition, 13 (8.8%) of the isolates were NT. Methicillin-resistant *S. aureus* (MRSA) strains constituted 33.1% (49/148) of the isolates. In the Irbid district, MRSA constituted 60.4% of *S. aureus* isolates compared with 20% of *S. aureus* isolates in Zarqa (p < 0.05) (Table-1). CP8 was predominant among all capsule genotype categories in both districts with no statistically significant difference. The distribution of capsule genotypes among MRSA strains was 79.6% for CP8 and 6.1% for CP5, and 14.2% were NT. Among the recovered capsule genotypes of *S. aureus* mastitis isolates, the majority of CP5 75% were MRSA compared with 29.8% in CP8 and 53.8% in NT. However, the statistical correlation could not be analyzed due to the low number of CP5 isolates recovered in this study.

**Table-1:** Distribution of capsule genotypes of *Staphylococcus aureus* isolates recovered by regions of the herd, species, and susceptibility to methicillin.

Region	Number of isolates (%)				

	All isolates	All isolates			

		MRSA	Cap 5	Cap 8	Nontypeable
Irbid	48 (32.4%)	29 (60.4%)	2 (6.9%)	21 (72.4%)	6 (20.7%)
Zarqa	100 (67.6%)	20 (20%)	1 (5%)	18 (90%)	1 (5%)
Total	148	49 (33.1%)	3 (6.1%)	39 (79.6%)	7 (14.2%)

MRSA=Methicillin-resistant *Staphylococcus aureus*, MSSA=Methicillin-susceptible *Staphylococcus aureus*

The capsule genotype distribution among methicillin-susceptible *S. aureus*/MRSA strains was not statistically significant (p > 0.05), and no correlation was established between the capsule genotypes and methicillin resistance.

### Antimicrobial susceptibility

The rates of antimicrobial resistance were 2.7% for C and CIP, 4.7% for SXT, 6.1% for FOX and CZ, 6.8% for DA, 7.4% for E, 10.1% for CN, and 22.3% for TE and DOX (Table-2). Furthermore, mastitis isolates from Irbid showed a significantly higher rate of resistance to CIP compared with those isolates from Zarqa district (8.3% vs. 0%), while Zarqa showed a significantly higher rate of resistance to CN compared with Irbid district (12.0% vs. 6.2%) (p < 0.05). CP5 isolates were only resistant to TE, DA, SXT, and DOX, with the same rate for each (25%). CP8 isolates showed resistance to all tested antibiotics at rates less than 11%, except for TE and DOX (Table-3). E and C resistance was only observed in CP8 isolates (8.4% and 3.1%, respectively) and was statistically significant for E (p < 0.05).

**Table-2:** Antimicrobial susceptibility profile of *S. aureus* isolates collected from milk samples of bovine mastitis and their distribution in relation to geographical districts.

Region	Frequency of antibiotic resistance among *S. aureus* mastitis isolates									

	Antimicrobial agent (quantity in ug/disc)									

	*CIP (5)	TE (30)	E (15)	DA (10)	SXT (25)	C (30)	CZ (30)	FOX (30)	DOX (30)	*CN (10)
Irbid (n=48)	4 (8.3)	11 (23)	5 (10)	3 (6.2)	4 (8.3)	1 (2.1)	4 (8.3)	4 (8.3)	11 (23)	3 (6.2)
Zarqa (n=100)	0	22 (22)	6 (6.0)	7 (7)	3 (3)	3 (3)	5 (5)	5 (5)	22 (22)	12 (12)
Total (n=148)	4 (2.7)	33 (22.3)	11 (7.4)	10 (6.8)	7 (4.7)	4 (2.7)	9 (6.1)	9 (6.1)	33 (22.3)	15 (10.1)
p-value	<0.05									<0.05

*S. aureus=Staphylococcus aureus*, CIP=Ciprofloxacin, TE=tetracycline, E=Erythromycin, DA=Clindamycin, SXT=Trimethoprim/sulfamethoxazole, C=Chloramphenicol, CZ=Ceftazidime, FOX=Cefoxitin, DOX=Doxycycline, CN=Gentamicin

**Table-3:** Frequency of antibiotic resistance in relation to capsule genotypes.

Antibiotics	CP5 (n=4)	%	CP8 (n=131)	%	NT (n=13)	%
CIP	0	0	4	3.1	0	0
TE	1	25	31	23.7	1	7.7
E*	0	0	11	8.4	0	0
DA	1	25	7	5.3	2	15.4
SXT	1	25	5	3.8	1	7.7
C	0	0	4	3.1	0	0
CZ	0	0	6	4.6	3	23.1
FOX	0	0	6	4.6	3	23.1
DOX*	1	25	31	23.7	1	7.7
CN	0	0	14	10.7	1	7.7

* Statistically significant at (p < 0.05). CP=Capsular polysaccharide, NT=Nontypeable, CIP=Ciprofloxacin, TE=tetracycline, E=Erythromycin, DA=Clindamycin, SXT=Trimethoprim/sulfamethoxazole, C=Chloramphenicol, CZ=Ceftazidime, FOX=Cefoxitin, DOX=Doxycycline, CN=Gentamicin

## Discussion

The prevalence of serotypes CP5 and CP8 among bovine mastitis isolates of *S. aureus* has been assessed in many countries and has revealed variability in their prevalence among countries. In our study, the majority of isolates possessed capsule genotypes (91.3%) with a predominance of CP8 (88.6%). Only 8.7% of the isolates were NT by PCR. In agreement with our results, several studies have shown a predominance of CP8. For instance, CP8 was reported to have a high prevalence in Denmark (23/39 isolates, 58.9%), Sweden (29/38 isolates, 76.3%) [21], Argentina (45/51 isolates, 88.2%), and Uruguay (21/38 isolates, 55.3%) [22]. In contrast, many other studies have reported CP5 as the dominant serotype in Argentina (83/150, 55.3%) [23], Brazil (128/159, 80%) [24], India (33/45 isolates, 73.3%) [25], Chile (37/55, 67.3%) [22], and Algeria (9/9, 100%) [26]. *Staphylococcus aureus* isolates that fail to produce CP5 or CP8 and produce nonmucosal colonies on solid media are known as NT strains, as defined by Cocchiaro *et al*. [27]. A study of 195 isolates conducted in Argentina by Sordelli *et al*. [15] demonstrated that only 13.8% could be typed by specific antisera against CP5 or CP8, while the remaining 168 isolates were nonreactive with CP5- or CP8-specific antibodies. According to O’Riordan and Lee [10], the expression of CP5 and CP8 is affected by both environmental signals and the *in vitro* environment. Therefore, it is possible that the culture conditions account for the difference in results observed in the previous studies [10]. In Jordan, a study conducted by Mattar *et al*. [28] on *S. aureus* strains isolated from humans revealed that CP5 had a higher prevalence, especially among MRSA strains, which contradicts our results of *S. aureus* isolated from bovine mastitis cases. As reported by Darwish *et al*. [29], this could be partially attributed to host–pathogen interaction of *S. aureus* infections and colonization potential and might be related to variations in clonal identity and dissemination of *S. aureus* isolates in Jordan. The results of Darwish *et al*. [29] showed the presence of fifteen different spa types in the target group of Jordanian patients, and there was no clear distribution correlation with the sources of colonization. Thus, it seems that colonization arises from multiple sources. Such a result could not be avoided in our study, despite sampling from more than one farm in different regions of Jordan.

Vaccines based on bacterial polysaccharides protect against many dangerous pathogens and save millions of lives annually [30]. Several trials have been undertaken to develop *S. aureus* CP5- and CP8-based vaccines, and a recent study by Zhao *et al*. [9] showed the great potential of *S. aureus* CP8 in vaccine development. The results showed a strong immune response to the chemical synthesis of conjugation-ready CP8 trisaccharide 1 in mice, demonstrating that synthetic trisaccharide 1 could be an effective antigen for vaccine development. The results of Zhao *et al*. [9] give a new horizon for researchers to continue the development of approved CP vaccines, particularly in light of the results of our current study in Jordan, where we discovered that most of the clinical isolates are capsule genotypes (91.3%) with a predominance of CP8 (88.6%). These findings provide hope for the development of a more effective vaccine for mastitis.

In the past decade, interest in antibiotic resistance to various bacterial pathogens has increased because it is a major public health concern due to the risk of transmission of resistance from animals and the environment to humans, as well as its impact on the efficacy of current antibiotics [31, 32]. Furthermore, *S. aureus* has become resistant to many commonly used antibiotics due to the growing use of antibiotics and the rapid development of MRSA strains [33]. MRSA infections are incredibly difficult to treat due to their multidrug-resistant characteristics and the expression of a wide range of virulence factors [34]. Our current study reported increased rates of MRSA strains (n = 49, 33.1%) among bovine mastitis cases. Interestingly, geographical variation was also of significance, and approximately two-thirds of MRSA strains were recovered from bovine mastitis cases in the Irbid district. Also, in Jordan, similar results were found in a previous study by Obaidat *et al*. [35], where milk samples collected from large milk tanks of cows, sheep, and goats showed a 26% prevalence of the *S. aureus* gene *mecA*. Specifically, MRSA was detected in 31.8% of cows, 29.8% of sheep, and 11.5% of goat dairy farms.

Our current study revealed that most of the *S. aureus* isolates were moderately resistant to TE, SO, and gentamicin and to a lesser extent to E, DA, FOX, CZ, SXT, C, and CIP. Indeed, comparing antibiotic resistance results between different studies is somewhat difficult. This may be due to differences in usage frequencies and long-term use, isolates from different sample types, and even between herds on the same farm. Generally, the isolates in our study showed lower resistance levels to the tested antibiotics than in previous studies in Jordan [35, 36]. This may be due to the variety of the milk sample sources, which were taken from large milk tanks in the studies of Obaidat *et al*. [35], Obaidat *et al*. [36], in comparison to our samples, which were collected directly from individual animals. The antimicrobial resistance results in our current study are slightly higher than the findings of Nam *et al*. [37] in Korea and somewhat lower than the results in other countries, such as Algeria [26], South Africa [38], and Egypt [39]. Notably, *S. aureus* strains collected from Irbid showed significant differences in resistance to CIP (p < 0.05) compared with those strains collected from Zarqa, which tended to be more resistant toward CN. It should be noted that farms in the Irbid district are small in size and not subject to veterinary supervision like the farms in Zarqa, so it is expected that the rate of misuse of antibiotics, such as CIP, to treat mastitis cases will be higher on farms in Irbid than those in Zarqa, which might explain this difference. In addition, increasing resistance toward TEs and DOX regardless of the region was reported. This is expected because of the misuse of these antibiotics on animal farms and the lack of antibiotic therapy protocols in the management of bovine mastitis in Jordan. Regarding capsule genotypes, associations with antimicrobial resistance were mostly not significant. Although CP8 strains showed resistance (either single or multiple) to all antimicrobial agents used in this study, the CP8 strains showed significant resistance to E, which was not observed in CP5 and NT strains (p < 0.05). However, due to the low number of CP5 strains isolated in our study, larger studies are necessary to confirm such a correlation.

## Conclusion

Capsule genotypes play a significant role in bovine mastitis and are partially related to variations in epidemiology and antimicrobial resistance of *S. aureus*. Thus, we recommend capsule genotyping of *S. aureus* to manage and control bovine mastitis in the area covered in our study as well as throughout Jordan. In addition, the presence of CP8 as a prominent serotype in Jordan may make it a suitable candidate for the development of a CP-based vaccine against *S. aureus*. The lack of access to more farms in other geographic areas of the country was the main limitation of our study. We believe that our study constitutes an important initiative to investigate the role and influence of capsule genotypes on both the spectrum of bovine mastitis and antibiotic resistance in our region. Future work should focus on monitoring antimicrobial resistance and antibiotic misuse in the veterinary field as well as its impact on human and public health.

## Authors’ Contributions

MHG and LFA: Conceptualization, Data curation, Project administration, and Writing – review and editing. LFA: Formal analysis and Writing – original draft. MHG: Methodology. Both authors have read and approved the final manuscript.
